# Early Pseudoprogression After Tarlatamab in Small‐Cell Lung Cancer: A Case Report

**DOI:** 10.1002/rcr2.70319

**Published:** 2025-08-17

**Authors:** Yoshio Nakano, Hiroka Serizawa, Yuri Enomoto, Yusuke Kuze, Norio Okamoto, Iwao Gohma

**Affiliations:** ^1^ Department of Respiratory Medicine Sakai City Medical Center Sakai Osaka Japan

**Keywords:** bispecific T‐cell engager therapy, pseudoprogression, small‐cell lung cancer, tarlatamab

## Abstract

Pseudoprogression, a transient radiographic flare caused by immune infiltration, is common after immune‐checkpoint inhibitors but has not been reported with tarlatamab, a bispecific T‐cell engager approved for third‐line small‐cell lung cancer (SCLC). A 57‐year‐old woman with extensive‐stage SCLC and syndrome of inappropriate antidiuretic hormone secretion (SIADH) received tarlatamab. Within hours, she developed bone pain; Day 7 imaging showed marked tumour swelling and pleural effusion despite negative cytology and rising serum sodium. Therapy continued. By Day 13, computed tomography demonstrated regression of thoracic and hepatic lesions and falling pro–gastrin‐releasing peptide (pro‐GRP). Early pseudoprogression and paraneoplastic biomarker improvement may predict efficacy.

## Graphical abstract

We describe a case of apparent disease worsening in a patient with small cell lung cancer shortly after the initiation of tarlatamab therapy; this was subsequently followed by rapid tumour regression, suggesting the occurrence of pseudoprogression.

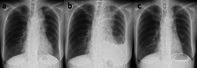

## 1 Introduction

Pseudoprogression, a phenomenon characterised by transient radiographic worsening due to immune cell infiltration rather than true tumour growth, has been increasingly recognised in the context of immunotherapies, such as checkpoint inhibitors, for non–small cell lung cancer (SCLC) [1]. Pseudoprogression has also been reported following immune checkpoint inhibitor (ICI) therapy in patients with SCLC [2]. Although rarely described, pseudoprogression is emerging as a clinically relevant consideration in bispecific T‐cell engager (BiTE) therapies, including agents such as epcoritamab and talquetamab, particularly in patients with lymphoma or multiple myeloma [3, 4]. This phenomenon most frequently occurs within a few days to 2 weeks after the initiation of BiTE therapy, typically presenting as a transient worsening on radiologic imaging, such as tumour enlargement. However, it is frequently followed by marked clinical or radiographic improvements, complicating the differentiation from true disease progression [3, 4].

Tarlatamab, a BiTE antibody, has shown clinical efficacy in previously treated SCLC [5]. However, pseudoprogression associated with tarlatamab has not been reported. Here, we describe a case of apparent disease worsening shortly after the initiation of tarlatamab therapy, which was subsequently followed by rapid tumour regression, suggesting the occurrence of pseudoprogression.

## 2 Case Report

A 57‐year‐old woman presented in July, Year *X* − 1, with back pain and was diagnosed with extensive‐disease SCLC (ED‐SCLC), cT3N2M1c, clinical stage IVB. Her serum sodium level at presentation was 117 mmol/L, consistent with the tumour‐related syndrome of inappropriate antidiuretic hormone secretion (SIADH). First‐line chemotherapy with carboplatin, etoposide, and durvalumab was immediately initiated. After four cycles, the patient achieved a partial response, but disease progression was documented following two cycles of maintenance durvalumab, characterised by multiple brain metastases and enlargement of both the primary lung lesion and extracranial deposits.

**FIGURE 1:**
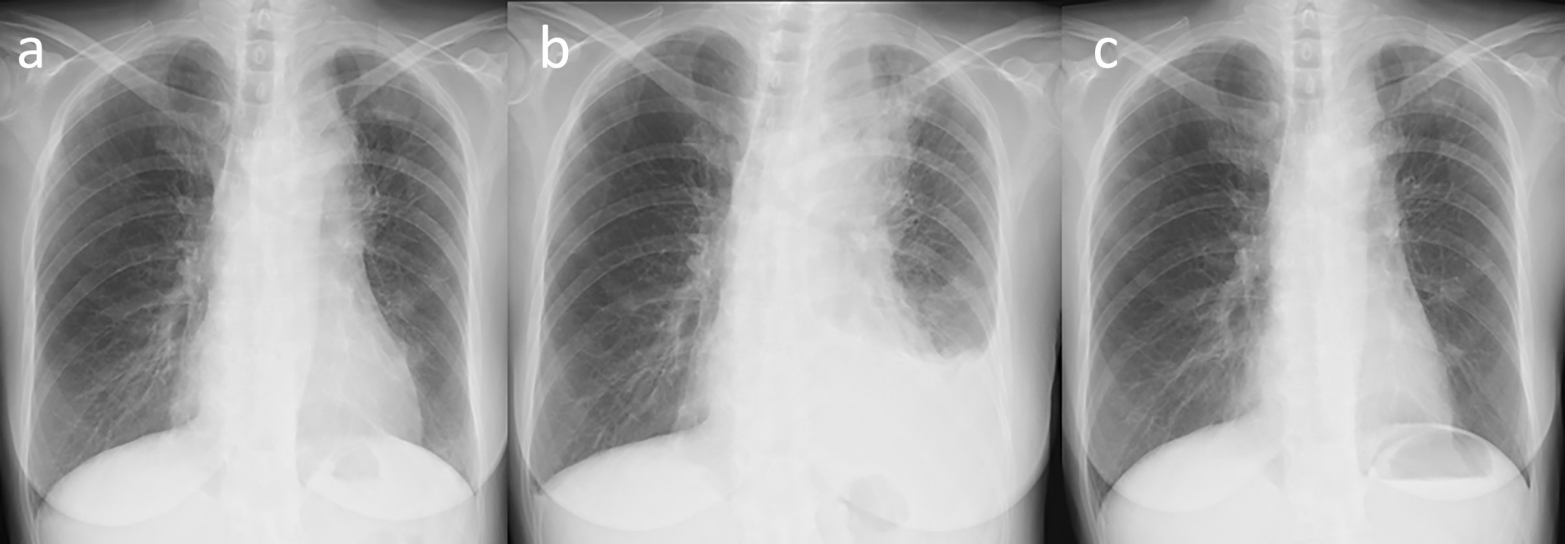
Serial chest radiographs before and after tarlatamab administration. (a) Baseline radiograph showing a left‐upper‐lobe mass without pleural effusion. (b) Day 7 after the first dose: Enlargement of the primary lesion and new left pleural effusion. (c) Day 12 after the first dose: The primary lesion is smaller than at baseline, and the pleural effusion has resolved.

**FIGURE 2:**
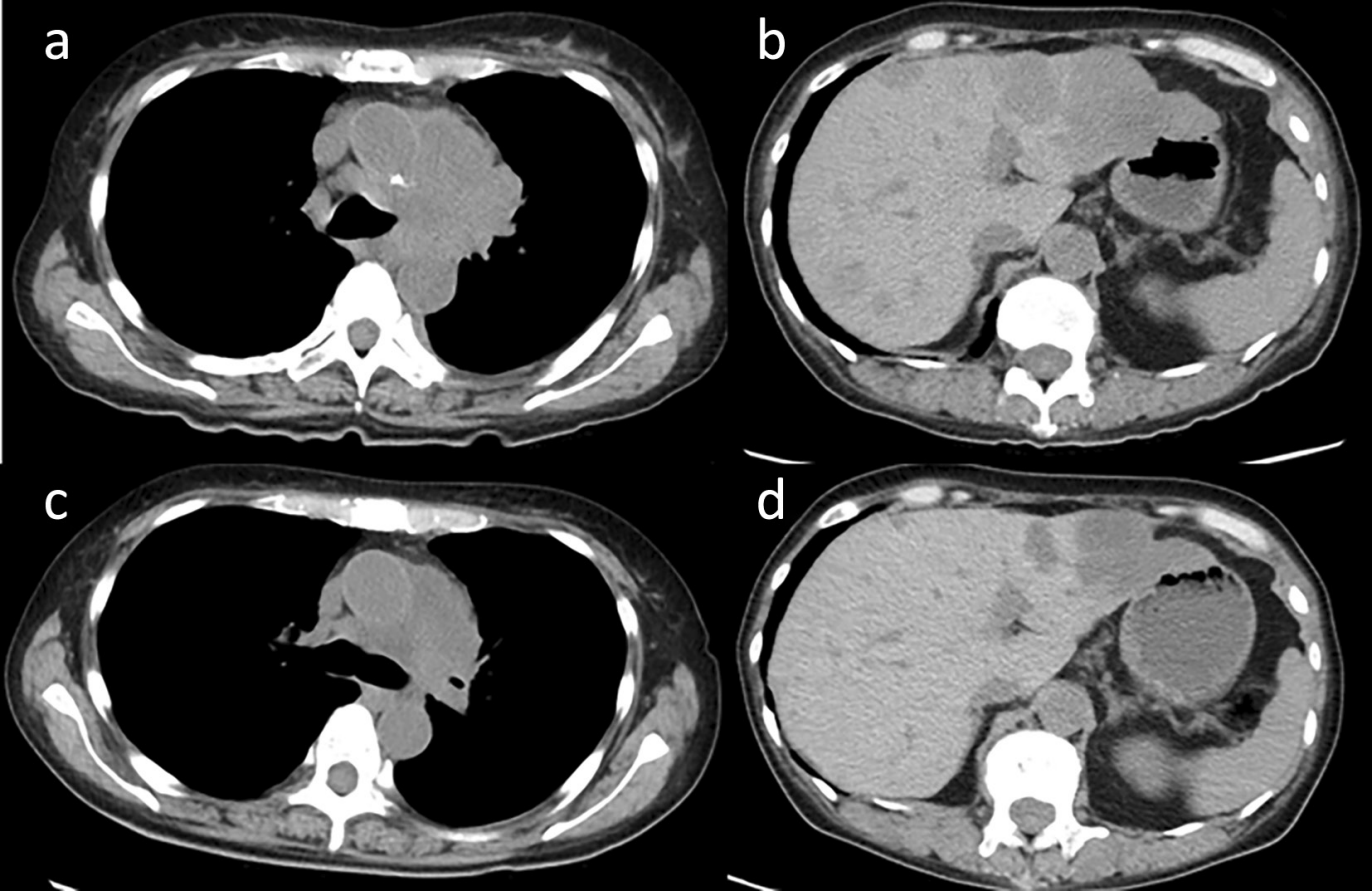
Chest computed tomography (CT) images before and after tarlatamab treatment. (a) Baseline CT demonstrating a mediastinal‐side mass in the left upper lobe. (b) Baseline CT demonstrating multiple hepatic metastases. (c) Day 13 post‐treatment: Marked reduction of the primary left upper lobe lesion. (d) Day 13 post‐treatment: Regression of hepatic metastases.

Second‐line treatment with amrubicin was initiated in December, Year *X* − 1. Although a partial response was observed after two cycles, generalised tumour regrowth resulted in progressive disease after six cycles (Figures 1a and 2a,b). Throughout the first‐ and second‐line treatments, the severity of SIADH fluctuated in parallel with the tumour burden, and opioid‐controlled bone pain developed.

Third‐line therapy with tarlatamab was initiated in May, Year *X*. Within several hours of the initial 1‐mg dose, the patient reported severe pain at the sites of spinal and pelvic metastases. At 11 h post‐administration, she developed a fever of 38°C. At that time, her vital signs were as follows: Japan Coma Scale (JCS) 0, heart rate 133 beats per minute, blood pressure 147/88 mmHg, respiratory rate 20 breaths per minute, and oxygen saturation (SpO_2_) 90% on room air. Given the presence of fever and hypoxaemia, Grade 2 cytokine release syndrome (CRS) was suspected. Tocilizumab was administered, resulting in the prompt resolution of both fever and hypoxaemia.

Because severe bone pain persisted despite opioid escalation, single‐fraction palliative radiotherapy (8 Gy) was administered to the cervical, thoracic, lumbar, and pelvic lesions on Day 5, resulting in rapid analgesia. However, chest radiography on Day 7 revealed marked enlargement of the primary tumour and a new left pleural effusion (Figure 1b). Thoracentesis showed no malignant cells. Concomitantly, the serum sodium level increased from 134 to 146 mmol/L, indicating SIADH improvement. The discordance between radiological worsening and biochemical improvement suggested tarlatamab‐induced pseudoprogression, and the treatment was continued.

**FIGURE 3:**
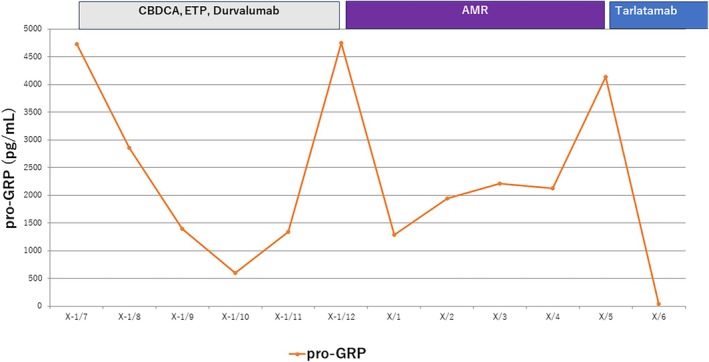
Timeline of systemic therapy and serial pro‐GRP levels. The serum pro‐gastrin‐releasing peptide (pro‐GRP) concentration mirrored the disease course; levels declined rapidly to the normal range after initiation of tarlatamab.

A 10‐mg dose was administered on Day 8. Transient bone pain recurred but was controlled with rescue opioids and NSAIDs and subsided by Day 9. Radiography on Day 12 demonstrated a reduction in both the primary lesion and pleural effusion (Figure 1c). These findings were confirmed by computed tomography on Day 13, which also showed regression of the hepatic metastases (Figure 2c,d). The absolute lymphocyte count in peripheral blood showed a decreasing trend, from 1091/μL before the administration of tarlatamab to 902/μL on Day 5 and 690/μL on Day 12 post‐treatment. This decline suggests the possibility of lymphocyte infiltration into the tumour tissue. Serum pro–gastrin‐releasing peptide levels decreased markedly, corroborating the tumour response (Figure 3). A subsequent 10‐mg dose on Day 15 was uneventful, and the patient continued outpatient tarlatamab with sustained clinical benefit.

## 3 Discussion

This case study provides two principal insights. First, SCLC patients treated with the BiTE antibody tarlatamab may exhibit immediate pseudoprogression following the initial infusion. Second, even when imaging or clinical findings suggest rapid tumour enlargement, tarlatamab should not be discontinued if there is concurrent evidence of antitumour activity, such as prompt improvement in paraneoplastic SIADH.

Pseudoprogression has been observed during the treatment of SCLC with the BiTE antibody tarlatamab.

Pseudoprogression is well documented in lung cancer receiving ICIs, yet remains rare in SCLC; only one isolated case following durvalumab has been reported [2]. In contrast, several BiTE antibodies, epcoritamab and talquetamab, have been associated with pseudoprogression in lymphoma and multiple myeloma [3, 4]. To date, no published reports have described this phenomenon with tarlatamab. Nevertheless, biopsy‐confirmed T‐cell infiltration during epcoritamab‐related pseudoprogression in diffuse large B‐cell lymphoma suggests a mechanistic rationale applicable to tarlatamab [3].

Tarlatamab is a novel therapeutic agent for SCLC that is currently approved only for use as a third‐line treatment; however, its clinical indications are expected to expand in the near future.

Our patient developed severe bone pain within hours of the first dose, accompanied by sudden pleural effusion and marked enlargement of the primary lesion. All symptoms regressed with continued therapy, confirming pseudoprogression. Because the patient's serum sodium level had previously mirrored disease activity, the rapid correction of SIADH after the first infusion further supported the ongoing drug efficacy. Clear communication about the likelihood of a treatment‐related flare enabled the patient to proceed with the third dose, after which the pain did not recur, and clinical improvement was sustained. The high baseline tumour burden may have amplified the immune‐mediated flare.

Tarlatamab precipitates early pseudoprogression in SCLC. When apparent disease worsening is observed shortly after the first dose, clinicians should consider pseudoprogression and, in the presence of objective markers of response (e.g., SIADH resolution), continue therapy while fostering a shared understanding with the patient.

## Author Contributions

Yoshio Nakano and Hiroyuki Hukuda conceived the study, prepared the data and drafted the manuscript. Yuri Enomoto and Iwao Gohma interpreted the data and revised the manuscript. Norio Okamoto and Yusuke Kuze supervised the study and reviewed the manuscript. All authors approved the final version of the manuscript.

## Ethics Statement

The authors declare that written informed consent was obtained for the publication of this manuscript and accompanying images and attest that the form used to obtain consent from the patient complies with the journal requirements as outlined in the author guidelines.

## Conflicts of Interest

The authors declare no conflicts of interest.

## Data Availability

The data that support the findings of this study are available on request from the corresponding author. The data are not publicly available due to privacy or ethical restrictions.
